# Fast growth rate of a right atrial myxoma

**DOI:** 10.31744/einstein_journal/2022RC6478

**Published:** 2022-03-17

**Authors:** Douglas Mesadri Gewehr, Alan Neiverth, Marcela Santos Cavalcanti, Thiago Ceschin Maestri, Semi Haurani, Fernando Bermudez Kubrusly, Luiz Fernando Kubrusly

**Affiliations:** 1 Faculdade Evangélica Mackenzie do Paraná Curitiba PR Brazil Faculdade Evangélica Mackenzie do Paraná , Curitiba , PR , Brazil .; 2 Neopath Patologia Diagnóstica Curitiba PR Brazil Neopath Patologia Diagnóstica , Curitiba , PR , Brazil .; 3 Unidade de Cardiologia Centro de Prevenção e Recuperação de Vidas Ltda Curitiba PR Brazil Unidade de Cardiologia , Centro de Prevenção e Recuperação de Vidas Ltda , Curitiba , PR , Brazil .; 4 Instituto Denton Cooley de Pesquisa, Ciência e Tecnologia Curitiba PR Brazil Instituto Denton Cooley de Pesquisa, Ciência e Tecnologia , Curitiba , PR , Brazil .

**Keywords:** Myxoma, Heart neoplasms, Heart atria, Dyspnea, Echocardiography

## Abstract

Primary cardiac tumors are rare, with an incidence between 0.0017 and 0.19%, and are asymptomatic in up to 72% of cases. Approximately 75% of tumors are benign, and nearly 50% of these are myxomas. Concerning location, 75% of myxomas are in the left atrium, 15 to 20% in the right atrium, and more rarely in the ventricles. The finding of cardiac myxomas usually implies immediate surgical excision to prevent embolic events and sudden cardiac death. Reports with documented growth rate are rare, and the actual growth rate remains a controversial issue. We report the rapid growth rate of a right atrial myxoma in an oligosymptomatic 69-year-old patient, with negative previous echocardiographic history in the last two years, who refused surgery upon diagnosis, enabling monitoring of myxoma growth.

## INTRODUCTION

Primary cardiac tumors are rare, with an incidence between 0.0017 and 0.19%, ^( 1 )^ and are asymptomatic in up to 72% of cases. Signs and symptoms of these neoplasms depend on their size, mobility and location, as well as physical activity and body position. The classic clinical manifestation is related to the Goodwin’s triad, which includes embolism, intracardiac obstruction and constitutional or unspecific systemic symptoms. ^( 2 )^ Approximately three quarters of the cardiac tumors are benign, and nearly half of these are myxomas. ^( 1 )^

Concerning location, 75% of myxomas are in the left atrium, 15 to 20% in the right atrium, and more rarely, in the ventricles. The occurrence in right atrium is more related to the male gender (4M:3F). ^( 1 - 4 )^

Myxomas are frequently excised soon after diagnosis, and because of that, their growth rate is generally unknown. ^( 1 - 5 )^ Just five cases of right atrial myxoma with documented growth rate have been reported in the medical literature, ranging from 0.07 to 1.36cm per month, with the first case reported only in 1994. ^( 6 - 10 )^

Very little is known about right atrial growth rate. In this report, our objective was to present the case of a rapidly growing right atrial myxoma, in an old female patient, with a previous echocardiographic history, who refused surgery upon diagnosis, enabling monitoring of myxoma growth.

## CASE REPORT

**Figure 1:**
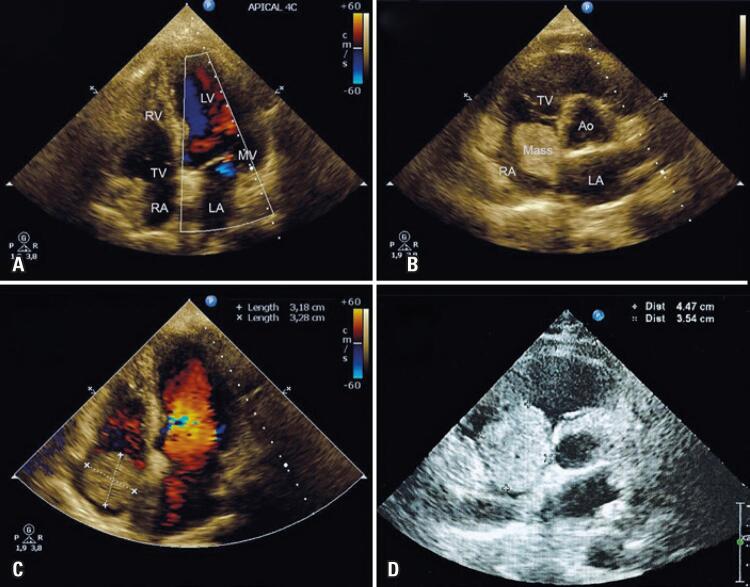
Echocardiography evolution of the right atrial mass. (A) Previous negative two-dimensional transthoracic echocardiogram showing apical four-chamber view and clear right atrium. (B and C) Diagnostic transthoracic echocardiogram (6 months later) showing aortic valve level (B) and apical four-chamber view (C), with a large homogenous right atrial mass attached on the atrial septum, measuring 3.18x3.28cm. (D) Pre-operative two-dimension transesophageal echocardiogram in mild-esophageal four-chamber view (6 months after diagnosis) demonstrating the rapidly growth of the right atrial mass, remaining attached on atrial septum and measuring 4.47x3.54cm

LV: left ventricle; MV: mitral valve; LA: left atrium; RA: right atrium; TV: tricuspid valve; RV: right ventricle; Ao: aorta.

A 69-year old female patient, in a routine annual visit to her cardiologist, complained of progressive dyspnea in the last month. She was classified as New York Heart Association (NYHA) Class II, with no alterations on her physical examination. Previous history of hypertension for 20 years, and moderate anemia. Her previous routine transthoracic echocardiogram (TTE), performed in the last four consecutive semesters, demonstrated a clear right atrium (Figure 1A). The diagnostic TTE, performed 6 months after the last negative TTE, showed a standard right atrium, with a 3.18x3.28cm homogenous mass attached to the atrial septum (Figures 1B and 1C). The rate of mass growth in the first 6 months was at least 0.530x0.546cm per month (1.74cm ^2^ per month).

**Figure 2:**
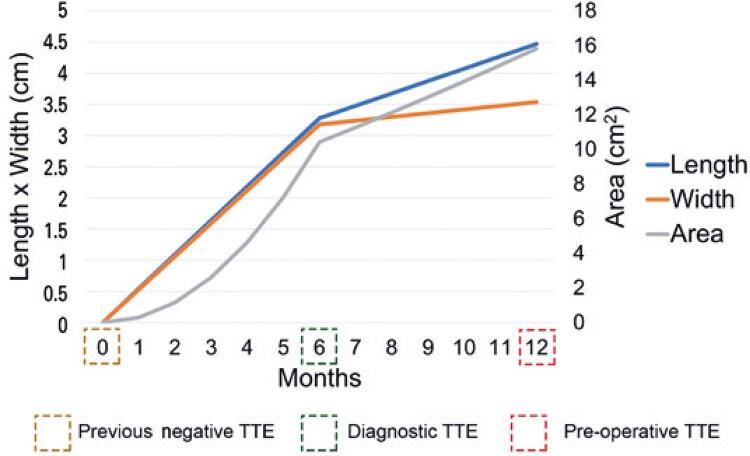
Right atrial myxoma growth rate over 12 months

Six months after the diagnosis, in a pre-operative consultation, a transesophageal echocardiography (TEE) showed a dilated right (34mL/m ^2^ ; reference: 27mL/m ^2^ ) and left atrium (43mL/m ^2^ ; reference: 34mL/m ^2^ ), with a 4.47x3.54cm homogenous right atrial mass, attached on the atrial septum (Figure 1D), reaching into tricuspid valve orifice. The growth rate after 6 months from diagnosis was 0.198x0.06cm per month (0.9cm ^2^ per month) ( Figure 2 ). The echocardiographies were performed by the same operator.

TTE: transthoracic echocardiogram.

**Figure 3:**
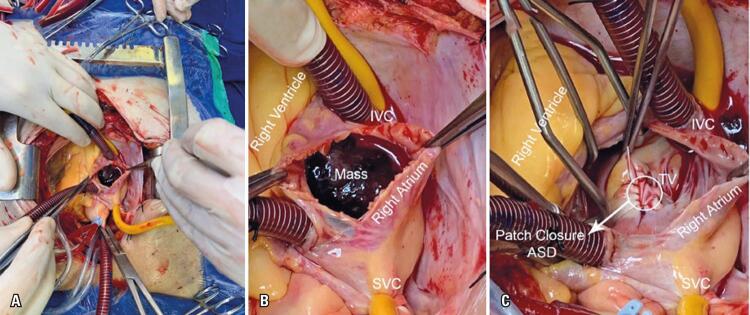
Illustration of the surgical excision right atrial myxoma. Operative picture showing arrested heart on cardiopulmonary bypass, with right atrial gelatinous lobulated mass *in situ* (A and B), immediately visualized after atriotomy. After myxoma excision, was performed a primary repair of atrial septal defect with patch closure (glutaraldehyde-treated bovine pericardium) (C)

IVC: inferior vena cava; SVC: superior vena cava; TV: tricuspid valve; ASD: atrium septal defect.

Our patient underwent elective cardiac surgery with complete excision of the right atrial mass, and its insertion in the atrial septum. The surgical approach was through a conventional median sternotomy and mediastinotomy. After anterior pericardiotomy, a bicaval cannulation was performed, followed by cardiopulmonary bypass and cardioplegic arrest. The right atrium was opened by an oblique incision, and the tumor was so large that it projected outside as the atrium was opened (Figures 3A and 3B). It was attached to atrial septum projecting into tricuspid valve orifice. The tricuspid valve was examined and found to be normal. The mass and the segment of its implantation were excised, including foci of tumor mass invading the left atrial surface of the atrial septum. The repair of the atrial septum defect (approximately 2cm in the fossa ovalis) was performed using bovine pericardium patch (Figure 3C). The heart returned into sinus rhythm, and cardiopulmonary bypass was slowly discontinued. The patient had an uneventful recovery.

**Figure 4:**
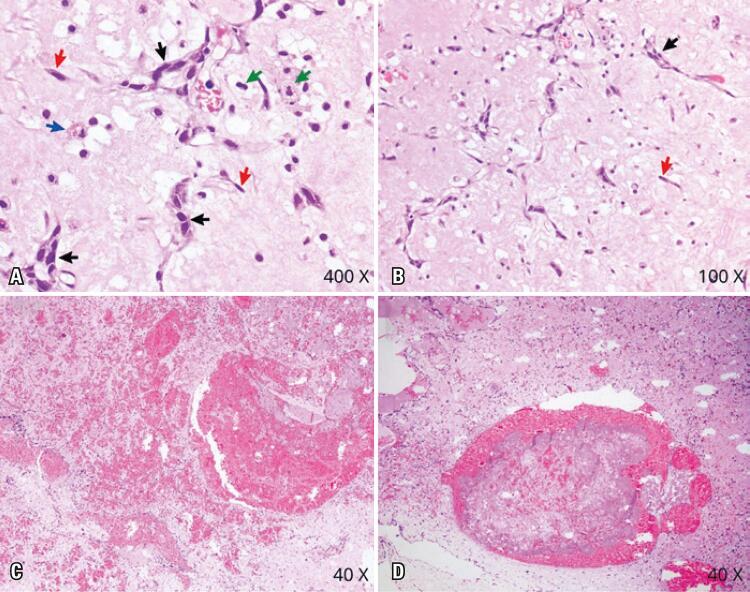
Representative histopathological fields from a portion of the myxoma. Multiple groups of aggregated/cord cells (black arrows), as well individual spindle cells (red arrows) in a myxomatous stroma (A and B), with recent and late hemorrhagic areas (C and D). The specimen had several areas of thrombosis (D). Degenerative cell atypia (green arrow), hemosiderin-laden macrophages (blue arrows), and plasma cells are found in several fields of this myxoma (hematoxylin and eosin A, 400x; B, 100x; C-D, 40x)

The macroscopic examination revealed the presence of multiple irregular fragments, brownish and translucent, soft and elastic, with a gelatinous aspect, weighing 19 grams and measuring 4.6x3.5x2.5cm. The histopathological examination revealed the presence of polygonal, spindle-shaped (lepidic) and stellate cells with the perivascular infiltrates in a myxomatous and hemorrhagic stroma suggestive of the myxoma (Figures 4A and 4B). The specimen had several areas of thrombosis of the intratumoral vessels, and areas of recent and late hemorrhage (Figures 4C and 4D). Degenerative cell atypia was also identified in some sites of the tumor (Figure 4A).

In the second postoperative month, the patient was considered NYHA Class I and there was no intracardiac mass on echocardiography.

This study was approved by the Research Ethics Committee of *Faculdade Evangélica Mackenzie do Paraná* under # 3.629.356, CAAE: 22132819.9.0000.0103.

## DISCUSSION

Myxomas account for 50 to 80% of cardiac neoplasms, with benign histological characteristics in most cases. They are usually seen in the left atrium (80 to 90%), with approximately 15 to 20% in the right atrium, and are most frequently attached to the interatrial septum. ^( 1 - 4 )^ Myxomas are generally asymptomatic, but may trigger some complications, such as functionally mitral and tricuspid stenosis, persistent anemia, syncope, heart failure, thromboembolic events, and even sudden cardiac death. ^( 1 - 5 )^ The most common clinical manifestation is dyspnea, ^( 3 )^ frequently associated with left atrial myxomas, as a sign of left heart failure, with NYHA Class III present in 61.3%, and NYHA Class II in only 20.9% of cases. ^( 11 )^ It is often related to tumor size. Tumor recurrence occurs in 3% of cases and the recurrence site is usually the original tumor site. ^( 2 )^

In this report, we described a case of right atrial myxoma, with a fast growth rate, found incidentally on a routine TTE. This a rare case, since it is the first to present images of right atrial myxoma at three time points, with six-month interval between echocardiograms. This was possible only because the patient refused surgical treatment upon first diagnosis, and had a previous negative echocardiogram, which enabled monitoring tumor growth over 12 months.

Despite the fast growth of the myxoma and its large size, the patient did not have a significant worsening of her clinical condition, characterizing a clinical-imaging dissociation.

In our case, no mass was apparent on the TTE performed 6 months prior to diagnosis, showing a growth rate of 1.74cm ^2^ per month (0.530x0.546cm per month), with a 3.18x3.28cm mass. In the pre-operative TEE, 6 months after diagnosis, the myxoma measured 4.47x3.54cm, implying a decrease in growth rate of the myxoma to 0.9cm ^2^ per month. The uncommon rapidly growth rate of the atrial myxoma in this patient prompted us to search the medical literature for other clinical reports on right atrial myxoma growth. We searched at MEDLINE ^®^ and PubMed Central ^®^ databases, with the descriptors “right and atrial and myxoma and growth”, and obtained a hit of 105 articles. Only clinical case reports with documented tumor growth of right atrial myxomas, with their calculated growth rate, were included in our analysis, and are summarized in table 1 . ^( 6 - 10 )^ Metastatic tumors, malign tumors, recurrent myxomas, review articles and other studies without documented growth rate were excluded.

**Table 1:** Published reports with documented right myxoma growth

Study	Age	Sex	Size at first echo	Size at diagnosis (cm)	Interval between echos (months)	Reported growth rate	Surgical removal at first diagnosis
Suzuki et al., ^(6)^	13	Male	No myxoma apparent	2.5x0.5	37	0.07x0.01cm/month 0.034cm ^2^ /month	No
Goldberg et al., ^(7)^	Early infancy	Female	No myxoma apparent	4.7x2.3	6	0.78x0.38cm/month 1.8cm ^2^ /month	No
Karlof et al., ^(8)^	58	Male	No myxoma apparent	15x3	11	1.36x0.3 cm/month 4.09cm ^2^ /month	No
Kelly et al., ^(9)^	71	Male	No myxoma apparent	5.8x5.3	12	0.48x0.44cm/month 2.5cm ^2^ /month	No
Heidari et al., ^(10)^	41	Female	No myxoma apparent	9x6	24	0.375x0.25cm/month 2.25cm ^2^ /month	No
Present study	69	Female	No myxoma apparent	3.28x3.18	6	0.546x0.530cm/month 1.74cm ^2^ /month	No

Most reports on cardiac tumor growth rates are related to left atrial myxomas, with a mean documented growth rate of 0.49cm per month, ^( 12 )^ still representing a minority of the reports. As from 1994, ^( 6 )^ only five cases of right atrial myxomas, with documented growth rate, were reported in medical literature. In fact, without serial echocardiography measurements, we are unable to delineate the growth pattern with accuracy. It is only possible in two occasions, when patient has heart disease history to follow-up by echocardiography, or when patient refuses surgery soon after diagnosis. In calculating these growth rates, it is assumed that myxomas grow in a linear fashion. ^( 8 - 12 )^

No malignancy or glandular degeneration was found on microscopic examination, which could imply a faster growth rate. Cardiac myxomas can show cell atypia, high cellularity or mitosis, without affecting the patient prognosis. ^( 13 , 14 )^ Therefore, the presence of degenerative atypia in our case, cannot be implied as a cause of increased myxoma growth rate. Thrombi are an important differential diagnosis of myxoma growth. They can be misinterpreted as myxoma growth. ^( 1 - 6 )^ Multiple foci of hemorrhage and thrombosis inside the myxoma may have been the cause of the rapidly increase in tumor volume, and anemia, in our patient.

## CONCLUSION

In summary, we report the case of a patient with a rapidly growing right atrial myxoma, with a growth rate of 1.74cm ^2^ per month. Because patient had a follow up with serial negative echocardiograms, and refused surgical treatment upon diagnosis, we were able to witness the growth of the tumor. Multiple foci of hemorrhage and thrombosis inside myxoma could imply in acceleration in its growth. Large atrial masses with this pattern of growth require surgical excision as early as possible, since they can evolve with thromboembolic events and sudden cardiac death, in addition to ruling out the possibility of malignant tumor. The potential for rapid growth of the intracardiac masses should be wisely considered if there is a plan to delay surgery or opt for a watchful waiting approach.
